# Phase I study of telatinib (BAY 57-9352): analysis of safety, pharmacokinetics, tumor efficacy, and biomarkers in patients with colorectal cancer

**DOI:** 10.1186/2045-824X-3-16

**Published:** 2011-07-29

**Authors:** Klaus Mross, Annette Frost, Max E Scheulen, Jürgen Krauss, Dirk Strumberg, Beate Schultheiss, Ulrike Fasol, Martin Büchert, Jörn Krätzschmer, Heinz Delesen, Prabhu Rajagopalan, Olaf Christensen

**Affiliations:** 1 Tumor Biology Center at the Albert-Ludwigs-University Freiburg, Germany; 2 West German Cancer Center, Essen, Germany; 3 Marienhospital Herne, University of Bochum, Herne, Germany; 4 Magnetic Resonance Development and Application Center, University Hospital of the Albert-Ludwigs-University Freiburg, Germany; 5 Bayer Schering Pharma, Berlin and Wuppertal, Germany; 6 Bayer Pharmaceuticals Corporation, Montville, NJ, USA

## Abstract

**Background:**

Telatinib (BAY 57-9352) is an orally available, small-molecule inhibitor of vascular endothelial growth factor receptors 2 and 3 (VEGFR-2/-3) and platelet-derived growth factor receptor β tyrosine kinases.

**Methods:**

In this multicenter phase I dose-escalation study including a phase II like expansion part, 39 patients with refractory colorectal cancer (CRC) were enrolled into 14 days on / 7 days off in repeating cycles of 28 days (n = 11) or continuous dosing groups (n = 28) to receive ≥ 600 mg telatinib twice-daily (bid).

**Results:**

Hypertension (28%) and diarrhoea (15%) were the most frequent study drug-related adverse events of CTC grade 3. In this population, no clear relationship between telatinib dose and individual C_max _and AUC was apparent in the 600 mg bid to 1500 mg bid dose range. No partial remission according to RECIST was reached, but 41% of the patients reached some tumour shrinkage during treatment. Tumour blood flow measured by dynamic contrast-enhanced magnetic resonance imaging and sVEGFR-2 plasma levels decreased with increasing telatinib AUC_(0-12)_.

**Conclusion:**

Telatinib treatment was well tolerated. The observed single agent antitumor activity in heavily pretreated CRC patients was limited. Pharmacodynamic results are suggestive for the biological activity of telatinib justifying a further evaluation of telatinib bid in combination with standard chemotherapy regimens in CRC patients.

## Background

Telatinib (BAY 57-9352) is an orally available, potent, small-molecule inhibitor of vascular endothelial growth factor (VEGF) receptors 2 and 3 (VEGFR-2/-3) and platelet-derived growth factor (PDGF) receptor β (PDGFR-β) tyrosine kinases. The data presented in this paper describe the results of colorectal cancer (CRC) patients from the dose escalation part and the phase II-like expansion part of a phase I dose escalation study in which patients received telatinib at doses of ≥ 600 mg bid (n = 39). Data from 19 of these 39 CRC patients were presented earlier [1].

## Methods

The study methods, treatment plan, patient evaluation, pharmacokinetic sampling, and pharmacodynamic measurements from the phase I dose escalation part of this study have been previously described [1] and the study assessments were not changed for the expansion part.

## Results

### Patient characteristics

A total of 39 Caucasian CRC patients (adenocarcinoma) were included into a noncontinuous (n = 11; telatinib administration 14 days on / 7 days off in repeating cycles of 28 days) and a continuous treatment group (n = 28; daily telatinib administration) at dose levels of 600 mg bid (overall n = 6; continuous n = 1), 900 mg bid (overall n = 6; continuous n = 3), 1200 mg bid (only continuous dosing n = 2), 1500 mg bid (overall n = 4; continuous n = 1), and 900 mg bid continuous dosing for the patients enrolled in the phase II-like extension cohort of the study (n = 21). Patient demographics are summarized in Table 1. The patients were heavily pretreated, 98% had received any prior systemic therapy: oxaliplatin (87%), fluorouracil (87%), irinotecan (77%), cetuximab (56%), capecitabine (28%), bevacizumab (15%), and mitomycin (15%).

**Table 1:** Patient demographics

Parameter	Non-continuous dosing (n = 11)	Continuous dosing (n = 28)	Total (n = 39)
Gender (female/male)	5 (45%)/6 (55%)	14 (50%)/14 (50%)	19 (49%)/20 (51%)
Mean age (range) (years)	62.4 (42-80)	59.4 (22-78)	60.2 (22-80)
Mean BMI (range) (kg/m^2^)	26.5 (21.9-32.0)	26.0 (18.2-37.4)	26.2 (18.2-37.4)
ECOG PS (0/1)	7 (64%)/4 (36%)	23 (82%)/5 (18%)	30 (77%)/9 (23%)
Prior chemotherapy	10 (91%)	28 (100%)	38 (97%)
Prior radiotherapy	3 (27%)	8 (29%)	11 (28%)
Prior surgery	11 (100%)	27 (96%)	38 (97%)

- BMI - body mass index

- ECOG PS - Eastern Cooperative Oncology Group performance status.

### Safety

The most frequent adverse event assessed by the investigators as study drug related was hypertension (all grades: 36%, grade 3: 28%). In 1 patient at 1500 mg bid noncontinuous dosing group, hypertension resulted in dose reduction and dose interruption. Other study drug related adverse events occurring in ≥15% of patients were diarrhoea (26%, grade 3: 15%), anorexia (26%, only grades 1 - 2), nausea (18%, only grades 1 - 2) and fatigue (15%, grade 3: 3%). Gastrointestinal toxicities occurred more often in the continuous dosing group (Table 2). There were no study drug-related adverse events of CTC grades 4 or 5 reported. Study drug-related adverse events leading to a dose reduction or study drug discontinuation followed by a restart were diarrhoea (n = 4), hypertension (n = 1) and nausea with vomiting (n = 1). Serious study drug-related adverse events occurred in 3 patients: diarrhoea, hypertension, hand-foot skin reaction and dehydration.

**Table 2:** Incidence of patients with treatment-emergent, study drug-related adverse events with worst CTC grade (all grades/grade 3)

NCI CTC term	Non-continuous dosing (n = 11)		Continuous dosing (n = 28)		Total (n = 39)	
	**All Grades**	**Grade 3**	**All Grades**	**Grade 3**	**All Grades**	**Grade 3**

Hypertension	7 (64%)	6 (55%)	7 (25%)	5 (18%)	14 (36%)	11 (28%)
Diarrhoea (patients with or without colostomy)			10 (36%)	6 (21%)	10 (26%)	6 (15%)
Anorexia	1 (9%)		9 (32%)		10 (26%)	
Nausea	1 (9%)		6 (21%)		7 (18%)	
Fatigue (lethargy, malaise, asthenia)	2 (18%)		3 (11%)	1 (4%)	6 (15%)	1 (3%)
Flatulence			4 (14%)		4 (10%)	
Vomiting	1 (9%)		3 (10%)		4 (10%)	
Voice changes/stridor/larynx (hoarseness)	3 (27%)		1 (4%)		4 (10%)	
Dizziness/lightheadedness			4 (14%)		4 (10%)	
Dyspepsia/heartburn			3 (11%)		3 (8%)	
Myalgia (muscle pain)	1 (9%)		2 (7%)		3 (8%)	
Weight loss			2 (7%)		2 (5%)	
Insomnia	1 (9%)		1 (4%)		2 (5%)	
Dehydration			1 (4%)	1 (4%)	1 (3%)	1 (3%)
Hand-foot skin reaction			1 (4%)	1 (4%)	1 (3%)	1 (3%)
Rash/desquamation			1 (4%)	1 (4%)	1 (3%)	1 (3%)
Platelets			1 (4%)		1 (3%)	
Palpitations			1 (4%)		1 (3%)	
Gastritis			1 (4%)		1 (3%)	
Stomatitis/pharyngitis (oral/pharyngeal mucositis)			1 (4%)		1 (3%)	
Taste disturbance (dysgeusia)			1 (4%)		1 (3%)	
Muscle weakness (not due to neuropathy)			1 (4%)		1 (3%)	
Neuropathy - sensory			1 (4%)		1 (3%)	
Headache			1 (4%)		1 (3%)	
Neuropathic pain (jaw, neuro, limb pain)			1 (4%)		1 (3%)	
Dry skin			1 (4%)		1 (3%)	

- NCI CTC - National Cancer Institute Common Toxicity Criteria.

### Pharmacokinetics

**Figure 1:**
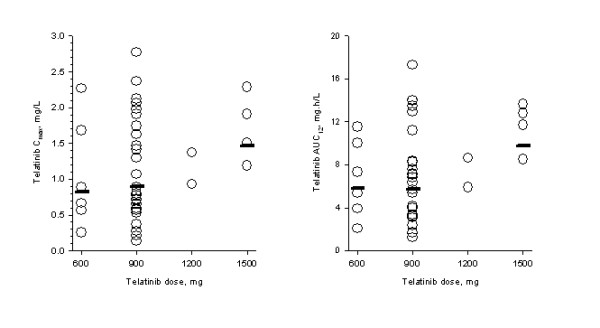
**Pharmakinetics.** Individual (open circles) and geometric mean (horizontal line) telatinib C_max _and AUC_(0-12) _on day 14 of cycle 1. Geometric mean for the combined 1200 mg plus 1500 mg dose levels is shown with 1500 mg data.

Steady-state pharmacokinetic parameters for telatinib and its metabolite BAY 60-8246 on day 14 of cycle 1 are summarized in Table 3. Maximum telatinib concentrations were typically observed less than 3 hours post-dose. No clear relationship between telatinib dose and C_max _and AUC was apparent in the 600 mg bid to 1500 mg bid dose range (Figure 1). Furthermore, average metabolite BAY 60-8246 C_max _and AUC values were also comparable in the 600 mg bid to 1500 mg bid dose range (Table 3).

**Table 3:** Geometric mean (% coefficient of variation) of telatinib and BAY 60-8246 pharmacokinetic parameters on day 14 of cycle 1

Parameter	Telatinib dose		
	600 mg bid (n = 6)	900 mg bid (n = 27)	1200 mg bid (n = 2) 1500 mg bid (n = 4) (n = 6)

Telatinib			
C_max _(mg/l)	0.825 (93%)	0.899 (91%) ^b^	1.467 (33%)
T_max _(h) ^a^	2.3 [1.1 - 4]	2.6 [0.5 - 8] ^b^	2.5 [0.67 - 4.1]
AUC_(0-tn) _(mg·h/l)	5.779 (71%)	5.547 (80%) ^b^	9.244 (31%)
AUC_(0-12) _(mg·h/l)	5.779 (71%)	5.761 (82%) ^b^	9.800 (33%)
Half-life (h)	8.2 (47%)	6.8 (47%) ^c^	8.1 (71%)

BAY 60-8246			
C_max _(mg/l)	0.101 (203%)	0.095 (120%)	0.207 (85%)
T_max _(h) ^a^	2.3 [0.6 - 4]	3.2 [0.5 - 12.2]	2.6 [0.5 - 4.1]
AUC_(0-tn) _(mg·h/l)	0.826 (181%)	0.634 (101%)	1.499 (93%)
AUC_(0-12) _(mg·h/l)	0.826 (182%)	0.636 (101%) ^d^	1.595 (92%)
Half-life (h)	6.2 (14%) ^d^	6.5 (48%) ^e^	7.8 (38%) ^b^

- AUC_(0-12) - _area under the plasma concentration versus time curve from time 0 to 12 hours

- AUC_(0-tn) - _area under the plasma concentration versus time curve from time 0 to last data point

- Bid - *bis in die*, twice daily

- C_max - _maximum plasma concentration

- T_max - _time to reach maximum plasma concentration.

a Median [range]

b Sample size reduced by 1

c Sample size reduced by 3

d Sample size reduced by 2

e Sample size reduced by 5.

### Pharmacodynamics

**Figure 2:**
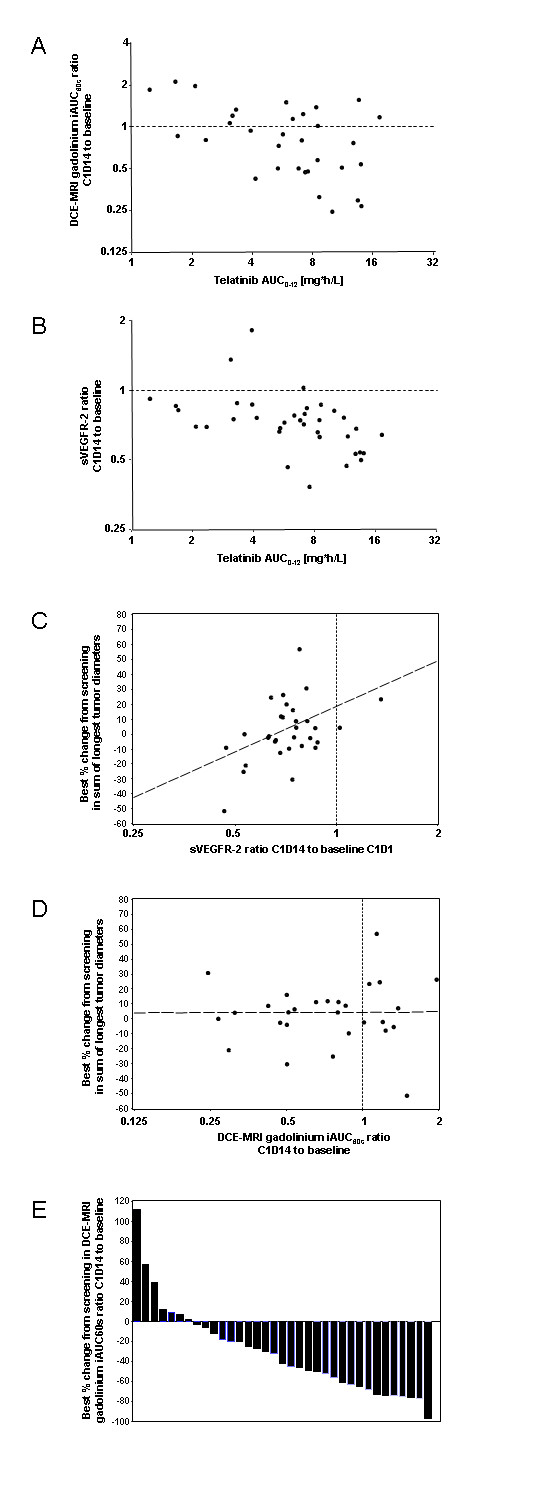
**Biomarkers, PK/PD. ** Analysis of telatinib AUC_(0-12) _on day 14 of cycle 1 versus the ratio of the initial 60 second area under the gadolinium curve (iAUC60) on day 14 of cycle 1 to the iAUC60 at baseline [A] and versus the ratio of sVEGFR-2 in plasma on day 14 of cycle 1 to sVEGFR-2 at baseline [B]; correlation of ratio of sVEGFR-2 in plasma on day 14 of cycle 1 to sVEGFR-2 at baseline to best % change from screening in sum of longest tumor diameters [C]; correlation of ratio of the DCE-MRI initial 60 second area under the gadolinium curve (iAUC60) on day 14 of cycle 1 to the iAUC60 at baseline to best percent change from screening in sum of longest tumor diameters [D]. Best percent change of the DCE-MRI initial 60 second area under the gadolinium curve (iAUC60) from screening compared to day 35 or any day later during study treatment. Each column is representing 1 patient [E].

The analysis of telatinib AUC_(0-12) _on day 14 of cycle 1 versus the ratio of the DCE-MRI contrast agent gadolinium iAUC60 on day 14 of cycle 1 to iAUC60 at baseline is shown in Figure 2a. The gadolinium iAUC60 ratio decreased with increasing telatinib AUC_(0-12) _(regression r^2 ^= 0.2637; Pearson correlation coefficient ρ = -0.514; test for no correlation, H_0_: ρ = 0, *p *= 0.0026).

The analysis of telatinib AUC_(0-12) _on day 14 of cycle 1 versus the ratio of sVEGFR-2 in plasma on day 14 of cycle 1 to sVEGFR-2 at baseline is shown in Figure 2b. The ratio of sVEGFR-2 decreased with increasing telatinib AUC_(0-12)_, i. e. essentially in an exposure-dependent manner (r^2 ^= 0.2241; ρ = -0.473; H_0_: ρ = 0, *p *= 0.0035).

In order to correlate biomarker changes to the clinical outcome, the relative changes between cycle 1, day 14 and baseline for VEGF, bFGF, tumour blood flow and tumour vessel permeability as measured by DCE-MRI (r^2 ^= 0.0025, ρ = 0.050, *p *= 0.7769; Figure 2d), and diastolic blood pressure were correlated to the best tumour shrinkage reached during therapy. The measured biomarkers were not predictive for the clinical outcome. However, the relative change between cycle 1, day 14 and baseline of sVEGFR-2 to the tumour shrinkage showed a tendency for correlation (r^2 ^= 0.2068, ρ = 0.455, *p *= 0.0102; Figure 2c). A decrease in tumour blood flow as measured by DCE-MRI was shown in the majority of patients (Figure 2e).

### Efficacy

**Figure 3:**
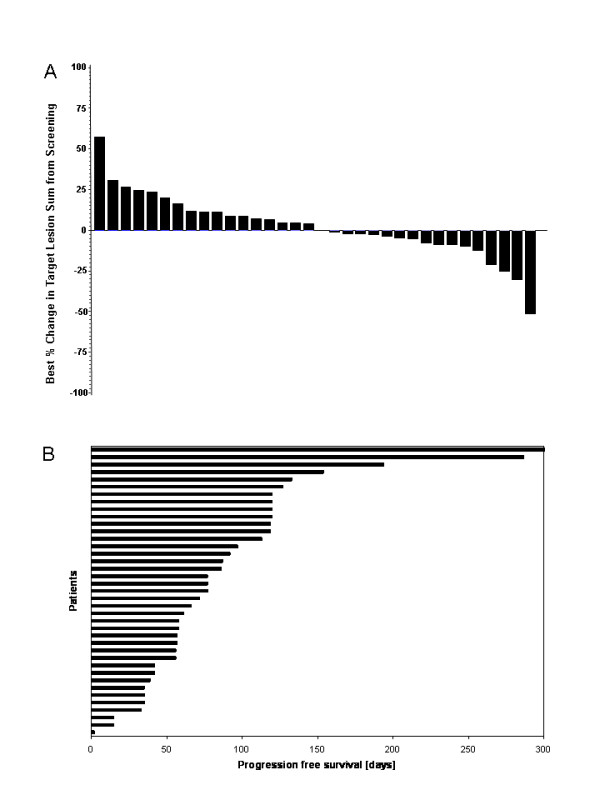
**Tumor efficacy.** Best percent change in target lesion sum from screening compared to day 35 or any day later during study treatment, each column is representing 1 patient [A]. Progression free survival (days), each column is representing 1 patient [B].

Tumour shrinkage at any point in time during treatment was observed in 41% of the patients (Figure 3a) and more than half had stable disease as best response (Table 4). The median progression free survival was 77 days (2 to 617 days) and 13 out of 39 patients. (33%) had a progression free survival of > 100 days (Figure 3b).

**Table 4:** Best response according to RECIST and treatment duration

Parameter	Non-continuous dosing (n = 11)	Continuous dosing (n = 28)	Total (n = 39)
Status at study entry (SD/PD)	1 (9%)/10 (91%)	0 (0%)/28 (100%)	1 (3%)/38 (97%)
Best response			
- Stable disease	6 (55%)	16 (57%)	22 (56%)
- Progressive disease measurement proven	4 (36%)	9 (32%)	13 (33%)
- Progressive disease by clinical judgment	1 (9%)	3 (11%)	4 (10%)

- PD - progressive disease

- SD - stable disease.

## Discussion

This analysis of 39 CRC patients enrolled in a phase I dose escalation study with a phase II like expansion cohort showed that telatinib administered at clinically relevant doses of ≥ 600 mg bid was well tolerated in this patient population. The recommended phase II dose for the single agent therapy with telatinib of 900 mg bid continuous dosing, as defined in the all comer dose escalation part of the study, was confirmed of being well tolerated in these heavily pretreated CRC patients. Hypertension was clinically manageable in most of the patients with a standard antihypertensive treatment. Study drug-related diarrhoea led to dose reduction or study drug discontinuation followed by a restart in 4 patients. The occurrence of gastrointestinal toxicities is known for other VEGF-inhibiting compounds [2,3].

The variability in pharmacokinetic parameters was considerable and individual patient telatinib exposure values were generally comparable in the dose range reported herein. Detailed pharmacokinetic analysis results in 71 patients covering a wider dose range of 75 mg bid to 1500 mg bid was reported earlier [1].

The biomarkers assessed in this study demonstrated the biological activity of telatinib. Most of the patients, 29 out of 36, showed a decrease of iAUC60 in the DCE-MRI measurements indicating an anti-angiogenic effect in tumour tissue. The angiogenic factors VEGF and sVEGFR-2 showed effects known from other VEGF-inhibiting compounds. Changes in the DCE-MRI and decreases in sVEGFR-2 were correlated to telatinib exposure. There was no correlation between the clinical outcome and the biomarker activity, only the correlation of sVEGFR-2 changes to the tumour shrinkage showed some dependency.

The treatment with single-agent telatinib showed no objective remission in patients with CRC refractory to standard chemotherapy. This is in line with phase II study results of single-agent sunitinib treatment in CRC patients [4]. However, one third of the CRC patients had a PFS of > 100 days, suggesting some clinical activity in this heavily pretreated patient population.

The profiles of all competitors are summarized in a review [5]. Telatinib is currently in the clinical development for Gastric carcinoma and showed promising results in a phase II study (ref.: J Clin Oncol 28, 2010 (suppl; abstr e14575), Ko et al.).

## Conclusions

The observed single agent antitumor activity of telatinib in heavily pretreated CRC patients was limited. Pharmacodynamic results are suggestive for the biological activity of telatinib. Further evaluation of telatinib bid in combination with standard chemotherapy regimens in CRC patients should be considered.

## Competing interests

JK, HD, PR and OC are full-time employees of Bayer. The authors declare no other competing interests.

## Authors' contributions

All authors contributed to the design of the study or manuscript writing, and have read and approved the final manuscript.
